# Temporal evolution of suicide by levels of rurality and deprivation among Japanese adults aged 20 years or over between 2009 and 2022

**DOI:** 10.1007/s00127-024-02718-x

**Published:** 2024-07-02

**Authors:** Eiji Yoshioka, Sharon J. B. Hanley, Yukihiro Sato, Yasuaki Saijo

**Affiliations:** 1 Department of Social Medicine, Asahikawa Medical University, Midorigaoka-higashi, 2-1-1-1, Asahikawa, Hokkaido 078-8510 Japan; 2 Department of Academic Primary Care, Institute of Applied Health Sciences, School of Medicine, Medical Sciences and Nutrition, University of Aberdeen, Foresterhill, AB25, 2ZB, Aberdeen, Scotland

**Keywords:** Suicide, COVID-19, Rurality, Socio-economic deprivation, Joinpoint regression, Japan

## Abstract

**Purpose:**

Previous studies have reported that levels of rurality and deprivation are factors associated with suicide risk. Reports on the association between rurality, deprivation and suicide incidence during the COVID-19 pandemic are scarce. The study aims to investigate how suicide rates evolved in areas with different levels of rurality and deprivation among Japanese adults aged 20 years or older between 2009 and 2022.

**Methods:**

This study used population density in 2020 as an indicator of rurality and per capita prefectural income in 2019 as a proxy for deprivation in Japan’s 47 prefectures. Joinpoint regression analysis was performed to analyze secular trends in suicide rates by rurality and deprivation.

**Results:**

Suicide rates for both men and women at different levels of rurality and deprivation remained roughly parallel during the research period. Suicide rates for men and women at all levels of rurality and deprivation were on a downward trend until around 2019, just before the onset of the pandemic. Following this, suicide rates in women showed a clear upward trend, while the trend in suicide rates for men also changed around 2019, with a slightly increasing or flat trend thereafter. Changes in suicide rates were greater among women and those aged 20–59 years.

**Conclusions:**

In Japan, time trends in suicide rates for both men and women have changed before and after the pandemic, but levels of rurality and deprivation across the 47 prefectures do not appear to have contributed much to these changes.

**Supplementary Information:**

The online version contains supplementary material available at 10.1007/s00127-024-02718-x.

## Introduction

The COVID-19 pandemic led to severe restriction on the daily lives of people globally. It has had a major impact on the mental health of populations, which may have increased suicide risk [1, 2]. However, based on previous reports, the impact of the pandemic on suicide risk is thought to be limited [3, 4]. One analysis of the number of suicides in 25 countries, including Japan, through December 2020 showed that the number of suicides significantly decreased in 11 countries post-pandemic compared to before, and only significantly increased in three countries, one of which was Japan [3]. A review of studies through July 2022 also reported that in most countries and regions suicides rates remained stable or decreased following the initial pandemic period [4]. The aforementioned multinational study and review mainly included high-income countries, with few results from low or middle income countries.

While previous studies have not shown an increase in suicides overall globally during the pandemic period, some studies have reported a marked increase in suicides among certain age groups or by gender [4, 5]. However, patterns were not consistent across countries or regions. In the Czech Republic and Austria, suicides increased only among males [3]. In China (Guangdong), Germany (Rhineland-Palatinate), Italy (Emilia-Romagna), and Taiwan, suicides increased among older adults (especially older men) [6–8]. In Japan, there was an increase in suicides in both genders and most age groups, but the increase was particularly pronounced among women and younger generation [3, 9].

Previous studies have reported that levels of rurality and deprivation are factors associated with suicide risk, and higher levels of rurality and deprivation tend to increase suicide risk [10, 11]. It is therefore possible that differences in levels of rurality and deprivation may play an important role in the evolution of suicides during the COVID-19 pandemic, however, studies investigating this association are limited [4, 5], and further research is needed from different countries and regions.

In Japan, there was a clear increase in suicides during the pandemic period compared to earlier periods, and this increase was particularly pronounced among women and the younger generation [9]. This situation in Japan is considered exceptional from a global perspective, but the reason for this remains unclear. While the demographic characteristics of the Japanese population most affected by the pandemic have been identified, the associations between rurality and deprivation have not been examined. To clarify this, we conducted a descriptive-epidemiological study to determine how suicide rates evolved in areas with different levels of rurality and deprivation before and after the onset of the pandemic in Japan.

## Methods

### Suicide and population data

We obtained data on the yearly number of suicides by gender, age group and prefecture of residence from the suicide statistics published by the Ministry of Health, Labor and Welfare in Japan [12]. The data we were able to obtain on the number of suicides by age group was divided into the following eight categories: under 20 years, 20 to 29 years, 30 to 39 years, 40 to 49 years, 50 to 59 years, 60 to 69 years, 70 to 79 years and 80 years or older. Because data on the number of confirmed yearly suicides was only available between 2009 and 2022, this period was used for the analysis. Supplementary Table 1 shows the number of suicides and the suicide mortality rate per 100,000 of the population by gender and age for the Japanese population in 2009 and 2022. As suicide rates among people under 20 are much lower than in other age groups, we did not use data from this age group in this study. Population data for each prefecture from 2009 to 2022 were based on population estimates published by the Statistics Bureau of Japan [13].

### Rurality and deprivation

This study used population density in 2020 as an indicator of rurality and per capita prefectural income in 2019 to estimate levels of deprivation in Japan’s 47 prefectures. Population density for each prefecture was obtained from the census in 2020 [14], and per capita prefectural income was obtained from prefectural accounts published by the Economic and Social Research Institute, Cabinet Office [15]. Forty-seven prefectures were grouped into tertiles based on population density and per capita prefectural income. Prefectures in the 1st tertile of population density are those with the highest levels of rurality and those in the 3rd tertile are those with the lowest levels of rurality. Similarly, the 1st tertile of per capita prefectural income represents the highest level of deprivation, whilst the 3rd tertile represent the lowest levels of deprivation. Maps of the distribution of Japan’s 47 prefectures, divided into tertiles of population density and prefectural per capita income, are shown in Supplementary Figures 1, 2, respectively. Descriptive statistics of tertiles of prefectural population density in 2020 and per capita prefectural income in 2019 in Japan are shown in Supplementary Table 2.

### Statistical analysis

This study investigates time series trends in age-adjusted suicide rates in years. Age-standardized suicide rates per 100,000 were calculated using the population structure in Japan in 2020 as the standard. To analyze secular trends in age-standardized suicide rates according to rurality and deprivation, we performed a joinpoint regression analysis, which allows for the identification of the calendar year (the joinpoint) in which statistically significant abrupt changes in temporal trends occurred [16]. A maximum of two joinpoints is allowed in the default setting since there are 14 time points of data in the study. 95% confidence intervals for a joinpoint were also calculated. Annual percentage change (APC) and the corresponding 95% confidence intervals were then calculated for each detected trend. All analyses were stratified by gender and age. Age groups were divided into four categories,: 20 years or older; 20-39 years; 40-59 years; and 60 years and over. We used the Joinpoint Regression Program, Version 5.0.1 (National Cancer Institute, Bethesda, Maryland, USA, http://surveillance.cancer.gov/joinpoint/). All other statistical analyses were performed using Stata statistical software, version 17.0, for Widows (StataCorp, College Station, TX, USA).

## Results

### Time trends in suicide rates by gender and age

**Fig. 1:**
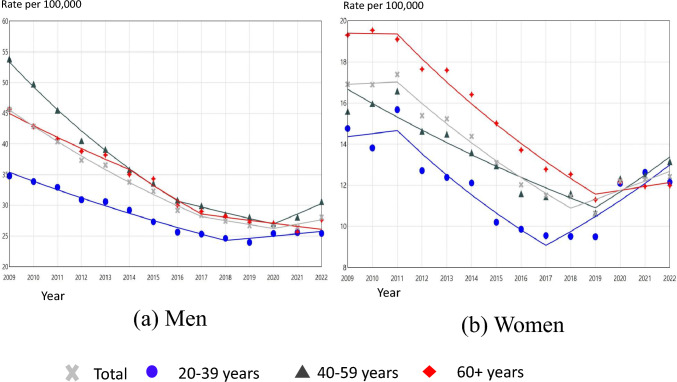
Age-standardized suicide rates per 100,000 population by age among **a** Japanese men **b** Japanese women, 2009–2022, with line segments from joinpointregression models

The total number of suicides in the study period for men aged 20 years and above was 230,635 (68.9%) and 104,331 for women (31.1%). The results of the Joinpoint regression analysis for men and women by age are shown in Fig. 1 and Table 1. The overall suicide rate for men showed a significant downward trend until 2020 (95% CI 2018 to 2020) and a statistically non-significant but increasing thereafter. Among all women, the suicide rate showed a significant downward trend from 2011 to 2018, and then a significant upward trend from 2018 (95% CI 2017 to 2019) onwards. The results by age showed that, for both men and women, the suicide rates in all three age groups followed roughly similar time trends over the study period, although there were slight differences by age. However, the differences in suicide rates for each age group for both men and women were much smaller in 2022 than in 2009.

**Table 1:** Summary of the Joinpoint regression analysis for trends in suicide rates by age, for Japanese men and women aged 20 or above, 2009–2022

	Segment 1			JP	(95% CI)	Segment 2			JP	(95% CI)	Segment 3			
	Period	APC	(95% CI)			Period	APC	(95% CI)			Period		APC	(95% CI)
Men														
Total	2009–2017	− 5.83*	(− 6.50 to − 5.51)	2017	(2011–2017)	2017–2020	− 2.36*	(− 5.94 to − 1.74)	2020	(2018–2020)	2020–2022		2.66	(− 0.19 to 4.39)
20–39 years	2009–2018	− 4.11*	(− 4.72 to − 3.66)	2018	(2017–2020)	2018–2022	1.49	(− 0.42 to 5.16)						
40–59 years	2009–2016	− 7.59*	(− 8.37 to − 7.13)	2016	(2014–2017)	2016–2020	− 3.21*	(− 5.66 to -1.26)	2020	(2019–2020)	2020–2022		6.02*	(2.08 to 8.33)
60 + years	2009–2014	− 4.43*	(− 4.93 to – 2.95)	2014	(2011–2014)	2014–2017	− 7.28*	(− 8.23 to – 5.67)	2017	(2016–2018)	2017–2022		− 1.80*	(− 2.71 to − 0.04)
Women														
Total	2009–2011	0.32	(− 3.81 to 4.55)	2011	(2011–2014)	2011–2018	− 6.18*	(− 8.40 to − 5.62)	2018	(2017–2019)	2018–2022		3.89*	(1.23 to 8.27)
20–39 years	2009–2011	1.02	(− 6.16 to 9.15)	2011	(2011–2016)	2011–2017	− 7.68*	(− 13.49 to − 0.66)	2017	(2016–2020)	2017–2022		7.45*	(3.45 to 14.50)
40–59 years	2009–2019	− 4.15*	(− 5.37 to − 3.31)	2019	(2017–2020)	2019–2022	7.05*	(1.71 to 15.69)						
60 + years	2009–2011	− 0.10	(− 4.31 to 4.47)	2011	(2011–2015)	2011–2019	− 6.23*	(− 9.50 to − 5.72)	2019	(2016–2020)	2019–2022		1.63	(− 2.43 to 7.29)

*APC* Annual percentage change, *JP* Joinpoint

*p-value < 0.05

### Time trends in suicide rates of men by prefectural levels of rurality and deprivation

**Fig. 2:**
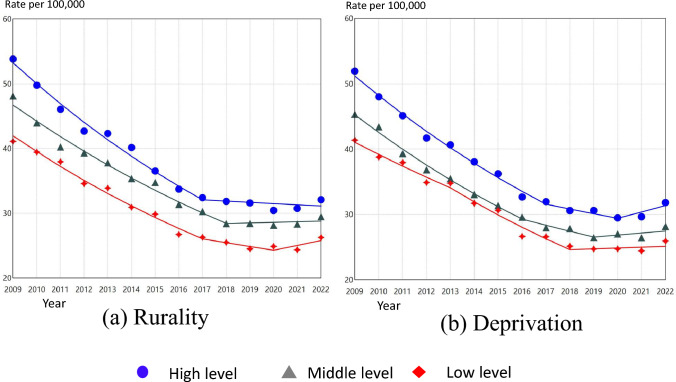
Age-standardized suicide rate per 100,000 population for Japanese men aged 20 years or above by rurality and deprivation, 2009–2022, with line segments from joinpointregression models

The results of the Joinpoint regression analysis for Japanese men aged 20 or older by prefectural levels of rurality and deprivation are presented in Figure 2 and Table 2. In areas with high levels of rurality, suicide rates in men decreased significantly until 2017 (95% CI 2014 to 2020) and have plateaued since. In the middle level of rurality, suicide rates showed a significant downward trend until 2018 (95% CI 2017 to 2019) and have plateaued since. In areas with low levels of rurality, a significantly decreasing trend in male suicide rates was observed until 2020 (95% CI 2018 to 2020), and then a non-significant but increasing trend. Regarding prefectural levels of rurality and male suicide rates between 2009 and 2022, the higher the rurality level, the higher the male suicide rate tended to be. And although suicide rates of all three levels remained roughly parallel throughout the study period, the difference in suicide rates between the levels was smaller in 2022 than in 2009.

**Table 2:** Summary of the Joinpoint regression analysis for trends in suicide rates by rurality and deprivation for Japanese men and women aged 20 or above, 2009–2022

	Segment 1			JP	(95% CI)	Segment 2			JP	(95% CI)	Segment 3		
	Period	APC	(95% CI)			Period	APC	(95% CI)			Period	APC	(95% CI)
Men													
Rurality^a^													
High	2009–2017	− 6.13*	(− 7.70 to − 5.30)	2017	(2014–2020)	2017–2022	− 0.61	(− 2.73 to 4.42)					
Middle	2009–2018	− 5.37*	(− 5.99 to − 4.89)	2018	(2017–2019)	2018–2022	0.31	(− 1.60 to 3.48)					
Low	2009–2017	− 5.78*	(− 6.68 to − 5.00)	2017	(2011–2017)	2017–2020	− 2.31*	(− 6.14 to − 1.54)	2020	(2018–2020)	2020–2022	3.04	(− 0.24 to 5.14)
Deprivation^b^													
High	2009–2017	− 5.86*	(− 7.18 to − 4.63)	2017	(2011–2017)	2017–2020	− 2.34*	(− 6.79 to − 1.42)	2020	(2017–2020)	2020–2022	3.27	(− 0.39 to 5.85)
Middle	2009–2016	− 6.02*	(− 7.30 to − 5.05)	2016	(2011–2017)	2016–2019	− 3.23*	(− 6.38 to − 1.01)	2019	(2017–2020)	2019–2022	1.14	(− 0.78 to 3.87)
Low	2009–2013	− 4.53*	(− 5.24 to − 2.54)	2013	(2011–2015)	2013–2018	− 6.30*	(− 8.01 to − 5.65)	2018	(2017–2019)	2018–2022	0.51	(− 0.85 to 2.89)
Women													
Rurality^a^													
High	2009–2018	− 5.59*	(− 6.30 to − 5.07)	2018	(2017–2019)	2018–2022	2.06	(− 0.48 to 6.22)					
Middle	2009–2011	0.27	(− 3.77 to 5.38)	2011	(2011–2015)	2011–2019	− 5.24*	(− 8.79 to − 4.71)	2019	(2018–2020)	2019–2022	6.78*	(1.91 to 14.77)
Low	2009–2011	1.65	(− 3.99 to 9.29)	2011	(2011–2014)	2011–2018	− 6.36*	(− 11.57 to − 5.30)	2018	(2016–2020)	2018–2022	4.40*	(0.38 to 13.91)
Deprivation^b^													
High	2009–2011	− 2.34	(− 6.00 to 2.23)	2011	(2011–2016)	2011–2018	− 6.04*	(− 9.47 to − 3.28)	2018	(2017–2020)	2018–2022	3.83*	(0.84 to 9.51)
Middle	2009–2011	− 0.04	(− 4.31 to 4.65)	2011	(2011–2015)	2011–2018	− 6.19*	(− 9.58 to − 5.35)	2018	(2016–2019)	2018–2022	3.30*	(0.35 to 8.46)
Low	2009–2011	2.19	(− 1.87 to 5.07)	2011	(2011–2012)	2011–2018	− 6.24*	(− 7.56 to − 5.64)	2018	(2017–2019)	2018–2022	4.31*	(2.02 to 7.63)

*APC* annual percentage change, *JP* joinpoint

*p-value < 0.05

^a^Rurality level was calculated based on the population density in 2020 of the 47 prefectures: the 1st tertile refers to high level, the 2nd refers to middle level, and the 3rd refers to low level

^b^Deprivation level was calculated based on the Per capita prefectural income in 2019 of the 47 prefectures: the 1st tertile refers to high level, the 2nd refers to middle level, and the 3rd refers to low level

In areas with high deprivation, suicide rates of men decreased significantly until 2020 (95% CI 2017 to 2020), after which they began a non-significant but increasing trend. In areas with medium level of deprivation, suicide rates decreased significantly until 2019 (95% CI 2017 to 2020), with a non-significant but slightly increasing trend thereafter. In areas with low levels of deprivation, suicide rates decreased significantly until 2018 (95% CI 2017 to 2019) and has plateaued since then. Suicide rates among Japanese men tended to be higher in prefectures with higher levels of deprivation during the study period. However, the difference in suicide rates between deprivation levels narrowed in 2022 compared to 2009.

### Time trends in suicide rates of women by prefectural levels of rurality and deprivation

**Fig. 3:**
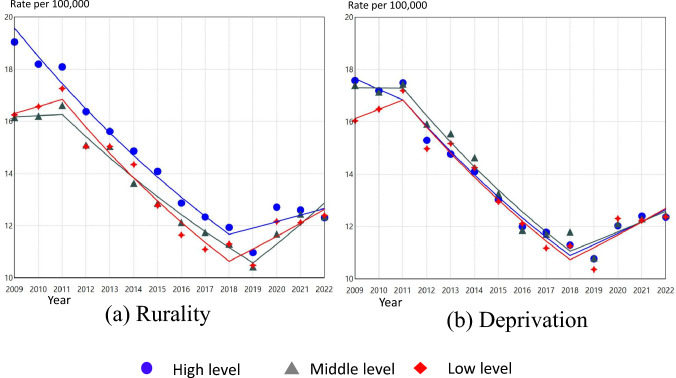
Age-standardized suicide rate per 100,000 population for Japanese women aged 20 years or above by rurality and deprivation, 2009–2022, with line segments from joinpointregression models

The results of the Joinpoint regression analysis for Japanese women aged 20 or older by prefectural levels of rurality and deprivation are presented in Figure 3 and Table 2. In areas with high levels of rurality, the suicide rate was on a significant downward trend until 2018 (95% CI 2017 to 2019) but has since turned to a non-significant but slightly increasing trend. In middle levels of rurality, suicide rates were on a significant downward trend from 2011 to 2019, then increased significantly from 2019 (95% CI 2018 to 2020) onwards. In areas with low levels of rurality, suicide rates were was on a significant downward trend from 2011 to 2018, then increased significantly from 2018 (95% CI 2016 to 2020) onwards. In 2009, suicide rates of women were higher in areas of high rurality, while the middle and low levels of rurality had almost the same level of suicide rates; in 2022, suicide rates between the three levels were almost the same.

Suicide rates of women were significantly decreasing from 2011 to 2018 for all three levels of deprivation, with a significant trend towards a significant increase from 2018. In 2009, the lowest level of deprivation had lower suicide rates than areas with low and medium levels of deprivation, but in 2022, suicide rates for all three levels were similar.

### Gender-/age-stratified results for time trends in suicide rates by prefectural levels of rurality and deprivation

Age stratified results of the Joinpoint regression analysis for Japanese men and women by levels of rurality and deprivation are shown in Supplementary Figures 3, 4, 5 and Supplementary Tables 3, 4, 5. For men aged 20–39 years, a significant increase in suicide rates was observed after 2017 in prefectures with high levels of rurality and deprivation. For women aged 20–39 years, a significant upward trend in suicide rates was observed from 2017 or 2018 onwards for all levels of rurality and deprivation. For men aged 40–59 years, a significant increase in suicide rates was observed after 2020 in areas with low levels of rurality and middle and low levels of deprivation. For women aged 40–59 years, a significant upward trend in suicide rates was observed from 2017 onwards in areas with low levels of rurality, and from 2017 to 2019 onwards in all levels of deprivation.

## Discussion

### Main findings of this study

In this study, we conducted a descriptive-epidemiological study using Joinpoint regression analyses to examine changes in suicide rates by prefectural levels of rurality and deprivation among Japanese adults aged 20 years or older from 2009 to 2022. The results showed that suicide rates in Japan for both men and women at different levels of rurality and deprivation remained roughly parallel during the research period. For both men and women, suicide rates at all levels of rurality and deprivation were on a downward trend until around 2019, just before the onset of the pandemic. And then, suicide rates in women showed a clear upward trend, while the trend in suicide rates of men also changed around 2019, with a slightly increasing or plateauing trend thereafter. For men, there were differences in suicide rates according to levels of rurality and deprivation, with suicide rates higher at higher levels of rurality and deprivation, although the differences in suicide rates were smaller in 2022 than in 2009. For women, on the other hand, there was little difference in suicide rates by levels of rurality and deprivation in 2022. Changes in suicide rates before and during the pandemic varied considerably by gender and age. The degree of change was greater for women than for men, and greater for those aged 20–59 years compared to those aged 60 years or older.

### Changes in suicide rates before and after the onset of the COVID-19 pandemic

The study examined trends in suicide rates broken down by gender, age, rurality and deprivation, and found that in most categories, around 2019 (the joinpoint was 2019 or the 95% CI for the joinpoint included 2019), the trend in suicide rates changed. That is, suicide rates were on a downward trend in all categories until about 2019, after which rates began to increase, level off, or the downward trend was mitigated. A previous study pointed to a marked change in suicide rates in Japan after 2020 compared to earlier years due to the COVID-19 pandemic [9], and the results of this study are also consistent with these results. However, not all groups by gender, age, rurality and deprivation had a trend change around 2019 in this study. There was a joinpoint in 2017 for some groups, but the 95% CI did not include 2019. For example, the time trend in suicide rates for men aged 60+ changed in 2017 from − 7.28% to − 1.80% for APC, and the 95% CI for 2017, which was a joinpoint, was from 2016 to 2018 and did not include 2019. This may indicate that changes in suicide rate trends were occurring in certain groups prior to the pandemic. Alternatively, it is possible that the joinpoint regression analysis used in this study could not have estimated the exact year of change. That is, in our analysis, Joinpoint regression analysis was analyzed with the default settings, which have limitations such as a maximum of two joinpoints in the research period and a minimum of two observation points between the joinpoints [17]. Furthermore, the study analyzed annual suicide rates, and data during the pandemic period are only available for three years, from 2020 to 2022. And thus, because there were not enough data points to analyze changes in suicide rates between, before, and after the onset of the pandemic, joinpoint analysis, although a widely used statistical analysis method to analyze changes in time-series trends, may not have adequately captured short-term changes in suicide rate trends in this study.

### Rurality and suicide

Previous studies indicated that rural rates of suicidal behavior and death by suicide were often higher than those in urban areas [18], but some studies have reported that the association between suicide risk and rurality/urbanicity varies significantly by gender and age [19–21]. The results of this study showed that in Japan from 2009 to 2020, suicide rates tended to be higher in rural areas than in urban areas for men, but there was little difference between rural and urban areas for women. There have been four reports on differences in the distribution of suicide rates between rural and urban areas during the pandemic, but these results are not consistent [22–25]. The greatest decrease in suicide rates in the USA during 2020 occurred in large metropolitan urban centres, whereas rates did not fall in the nation’s predominantly rural regions [22]. Higher population density predicted suicide rate increases across Mexico’s 32 states in 2020, with approximately twice as many suicides occurring in Mexico City than the expected value [23]. The national suicide rate in Brazil fell by 13% between March and December 2020, but substantial excess suicide risks were observed in some gender and age groups in states with greater population density [24]. In Ecuador, the rate of suicides occurring in urban and coastal areas increased [25]. The results of this study show that suicide rates at three different levels of rurality for both men and women remained roughly parallel before and after the onset of the pandemic, which suggests that differences in prefectural rurality levels do not seem to contribute much to the increase in suicide rates during the pandemic. However, on closer inspection, it is possible that, for both men and women, there is a slightly stronger tendency for suicide rates to increase during the pandemic period in prefectures with lower levels of rurality than in those with higher levels. This may indicate that the impact of the pandemic on suicide risk has been slightly stronger in prefectures with lower levels of rurality in Japan. The association between suicide and rurality during the pandemic in Japan will need to be studied further.

### Deprivation and suicide

Previous articles have found relatively strong evidence for positive associations between area-level socioeconomic deprivation and suicidal behavior among men but weaker evidence for women [11, 26]. A study using data on suicides in Japanese municipalities between 2009 and 2017 found an association between social deprivation and suicide risk, but it was more pronounced among men [27]. The results of this study showed that in Japan from 2009 to 2020, suicide rates tended to be higher in socioeconomically only deprived and less deprived areas for women. A finding that the COVID-19 pandemic has shown a disproportionate socioeconomic impact on suicide rates have so far come from India [28]. The research examining trends in suicide rates in India across regions with different socioeconomic status found that the increase was fivefold higher among males residing in states with high deprivation. A review article of research up to July 2022 indicated that evidence from some countries and regions partially supported the potential role of socioeconomic disadvantage as an important actionable effect modifier of the association between pandemic-related stressors and suicide [4]. The results of this study show that suicide rates at three different levels of deprivation for both men and women remained roughly parallel before and after the onset of the pandemic, which suggests that differences in prefectural deprivation levels do not seem to contribute much to the increase in suicide rates during the pandemic. However, on closer inspection, it is possible that, for men only, there is a slightly stronger tendency for suicide rates to increase during the pandemic period in prefectures with higher levels of deprivation than in those with lower levels. This may indicate that the impact of the pandemic on suicide risk has been slightly stronger in more socioeconomically deprived prefectures among Japanese men. The association between suicide and area deprivation during the pandemic in Japan will need to be studied further.

### Suicides by age

The results of this study also showed that during the research period there were differences in suicide rates by age, especially after about 2019, with distinct differences between those aged 20–59 years and over 60 years of age. That is, a trend in the suicide rates was changing for both the 20–59 years and the 60 years or older age groups around 2019, but the change appeared to be much smaller for the 60 years or older age group. The previously mentioned review article indicated that differences in risk of COVID-19 infection might have an effect on the distribution of area suicide risk [4]. That is because, in some countries and regions, suicide rates among older people, especially older men, increased disproportionately during the pandemic, possibly due to increased fear of infection and death, loss of partners and close friends, and loneliness due to isolation; stressors that affect older adults more than their working-age counterparts. However, this study showed that while the pandemic may have altered suicide rates among the elderly in Japan, this effect was considerably smaller than among younger generations. Therefore, the impact of differences in risk of COVID-19 infection on people’s suicide risk in Japan would not be as substantial.

The results of this study showed that suicide rates increased during the pandemic among men aged 40 to 59 years. This is something that was not emphasized much in a previous report in Japan [9]. This group had an increased suicide rate in 2022 (30.6 per 100,000) compared to 2021 (28.1 per 100,000). Previous reports from Japan have been up to 2021, and thus they would have not detected an increase in this gender-age group. Men of middle-aged are considered a group at comparatively high risk of suicide in times of economic recession in previous studies [29]. The result of this study may be an indication that the pandemic has lasted long enough, and the socioeconomic situation has deteriorated, gradually taking its toll on this gender-age-group in Japan.

### Limitations

The study had several limitations that deserve discussion. First, analyses are based on data from suicide statistics compiled by the police agency based on data on unnatural deaths, which might underestimate true suicide rates. However, the focus of this study is not on the extent of suicide rates, but rather on the time trends in suicide rates. As there has been no change in the way data are collected by the police over the period of this study, we believe that if there has been an underestimation of the number of suicides, the impact may not be substantial. Second, this study used prefectures as the geographical unit, which is a comparatively large unit. And thus, deprivation assessed by average income and rurality assessed by population density may not adequately reflect the situation of many of the residents. Consequently, it will be necessary in the future to conduct analyses on a smaller area unit or using data from individuals. Third, since the study was stratified by gender and age group, some categories may not have had sufficient power to detect statistically significant changes, even if the APC values were of some magnitude. Fourth, for technical reasons, this study was unable to present data at the prefectural level specifically from those 10–19 years-of-age. This means that the results are not fully reflective of the Japanese population as a whole. In addition, supplementary table 1 shows that suicide rates for most age groups in Japan decreased in 2022 compared to 2009. However, for the under-20 age group, rates increased for both men and women. This suggests that although suicide rates are considerably lower for people under 20-years-of-age than for other age groups, from a public health perspective, they are a group for whom suicide prevention should be a priority. Thus, the recent increase in suicide rates in those under 20-years-of-age in Japan needs to be addressed in further studies. Fifth, this study assessed the level of deprivation in each prefecture based on prefectural per capita income data for 2019, just before the pandemic. While levels of deprivation in individual prefectures may have changed substantially since the pandemic, this could not be taken into account in this study. Last, the study was descriptive in design, and the delineation of the complex relationships among risk factors of suicide was beyond the scope of this study.

### Implications for public health policies and future research

The findings of this study showed that the decreasing trend in suicide rates among Japanese adults aged 20 years or older before the onset of the COVID-19 pandemic was followed by a substantial change in the trend after the onset of the pandemic. This may indicate that support for the needy has not been successful in Japan because adequate employment support and aggressive fiscal spending may prevent an increase in suicides during economic downturns [30]. And thus, future research will be needed to investigate the impact of financial and employment support initiatives on people’s mental health and suicide risk in Japan during or after the pandemic. In addition, as of September 2023, Japan continues to see new cases of COVID-19 infections, but few restrictions to daily life have been put in place as a measure to combat infection. However, the number of suicides in Japan will need to be monitored carefully in the future, as there may be ongoing adverse impacts on socio-economically vulnerable populations, even today.

The results of this study did not provide clear evidence that the level of rurality or deprivation of the area affected the evolution of suicide rates during the pandemic in Japan. However, the effect of the pandemic on suicide risk may have been somewhat greater for men and women in rural areas and for men in deprived areas, and further studies with more detailed data, such as mortality data for smaller geographical units used in this study or individual mortality data, are needed. If it were to be shown that the impact of a pandemic and its associated economic impact and restrictions on daily life on people’s suicide risk varied according to levels of rurality and deprivation, this would be an important finding for suicide prevention in the next pandemics and major economic shocks in the future.

## Conclusion

The secular trend of suicide rates among Japanese adults aged 20 years or older has changed significantly after 2020 compared to before 2019 across gender, age groups, and prefectural rurality and deprivation levels. This change in suicide rates can be due to changes in social conditions following the COVID-19 pandemic since 2020. The extent of change in suicide rates did not appear to vary much between prefectural levels of rurality and deprivation. However, there were clear differences by gender and age group. That is, the degree of change was greater for women than for men, and the degree of change was greater for those aged 20–59 years than for those over 60 years. Only a few high-income countries reported an increase in the number of suicides during the pandemic, and a substantial increase in suicides in Japan is quite exceptional globally. And thus, further research into the background to the increase in suicides during the pandemic in Japan will be needed to establish appropriate suicide prevention measures.

## Supplementary Information

Below is the link to the electronic supplementary material.

Supplementary file1 (PDF 821 KB)

Supplementary file2 (PDF 261 KB)

## Data Availability

All data used in this manuscript are publicly available. Suicide data are available from the website of suicide statistics (https://www.mhlw.go.jp/stf/seisakunitsuite/bunya/0000140901.html). Data about population estimate are available from the website of Statistics Japan (https://www.e-stat.go.jp/en/stat-search/files?page=1&layout=datalist&toukei=00200524&tstat=000000090001&cycle=1&tclass1=000001011678&cycle_facet=tclass1%3Acycle&tclass2val=0). Data about Census in 2020 are available from the website of Statistics Japan (https://www.e-stat.go.jp/en/stat-search/files?page=1&toukei=00200521&tstat=000001136464). Data about per capita prefectural income in 2019 are available from the website of Statistics Japan (https://www.esri.cao.go.jp/jp/sna/data/data_list/kenmin/files/contents/main_2019.html).

## Code availability

Statistical analyses were performed using the Joinpoint Regression Program, Version 5.0.1 (National Cancer Institute, Bethesda, Maryland, USA, http://surveillance.cancer.gov/joinpoint/) and Stata statistical software, version 17.0, for Widows (StataCorp, College Station, TX, USA). All maps were produced using QGIS Version 3.22.13 for windows.

## References

[1] Pierce M, Hope H, Ford T et al (2020) Mental health before and during the COVID-19 pandemic: a longitudinal probability sample survey of the UK population. Lancet Psychiatr 7:883–892. 10.1016/S2215-0366(20)30308-4 10.1016/S2215-0366(20)30308-4 PMC7373389 32707037

[2] Gunnell D, Appleby L, Arensman E et al (2020) Suicide risk and prevention during the COVID-19 pandemic. Lancet Psychiatr 7:468–471. 10.1016/S2215-0366(20)30171-1 10.1016/S2215-0366(20)30171-1 PMC7173821 32330430

[3] Pirkis J, Gunnell D, Shin S et al (2022) Suicide numbers during the first 9–15 months of the COVID-19 pandemic compared with pre-existing trends: an interrupted time series analysis in 33 countries. EClinicalMedicine 51:101573. 10.1016/j.eclinm.2022.101573 35935344 10.1016/j.eclinm.2022.101573 PMC9344880

[4] Martínez-Alés G, Szmulewicz A, López-Cuadrado T et al (2023) Suicide following the COVID-19 pandemic outbreak: variation across place, over time, and across sociodemographic groups. a systematic integrative review. Curr Psychiatry Rep 25:283–300 37227647 10.1007/s11920-023-01427-7 PMC10209574

[5] Webb RT, John A, Knipe D et al (2022) Has the COVID-19 pandemic influenced suicide rates differentially according to socioeconomic indices and ethnicity? More evidence is needed globally. Epidemiol Psychiatr Sci 31:e72 36217667 10.1017/S2045796022000543 PMC9579839

[6] Zheng XY, Tang SL, Ma SL et al (2021) Trends of injury mortality during the COVID-19 period in Guangdong, China: a population-based retrospective analysis. BMJ Open 11:e045317. 10.1136/bmjopen-2020-045317 34083336 10.1136/bmjopen-2020-045317 PMC8182756

[7] Wollschläger D, Schmidtmann I, Blettner M et al (2021) Suicides during the COVID-19 pandemic 2020 compared to the years 2011–2019 in Rhineland-Palatinate (Germany) and Emilia-Romagna (Italy). Dtsch Arztebl Int 118:814–815. 10.3238/arztebl.m2021.0365 35191372 10.3238/arztebl.m2021.0365 PMC8884071

[8] Chen YY, Yang CT, Pinkney E, Yip PSF (2022) Suicide trends varied by age-subgroups during the COVID-19 pandemic in 2020 in Taiwan. J Formos Med Assoc 121:1174–1177. 10.1016/j.jfma.2021.09.021 34674903 10.1016/j.jfma.2021.09.021 PMC8493279

[9] Yoshioka E, Hanley SJB, Sato Y, Saijo Y (2022) Impact of the COVID-19 pandemic on suicide rates in Japan through december 2021: an interrupted time series analysis. Lancet Reg Health West Pac 24:100480. 10.1016/j.lanwpc.2022.100480 35655718 10.1016/j.lanwpc.2022.100480 PMC9150875

[10] Rehkopf DH, Buka SL (2006) The association between suicide and the socio-economic characteristics of geographical areas: a systematic review. Psychol Med 36:145–157 16420711 10.1017/S003329170500588X

[11] Cairns JM, Graham E, Bambra C (2017) Area-level socioeconomic disadvantage and suicidal behaviour in Europe: a systematic review. Soc Sci Med 192:102–111 28965001 10.1016/j.socscimed.2017.09.034

[12] Ministry of health, labor and welfare, Japan (2023) Suicide statistics [in Japanese]. https://www.mhlw.go.jp/stf/seisakunitsuite/bunya/0000140901.html. Accessed 4 Oct 2023

[13] Statistics of Japan (2023) Population estimates in Japan. https://www.e-stat.go.jp/en/stat-search/files?page=1&layout=datalist&toukei=00200524&tstat=000000090001&cycle=1&tclass1=000001011678&cycle_facet=tclass1%3Acycle&tclass2val=0. Accessed 4 Oct 2023

[14] Ministry of internal affairs and communications, Japan (2022) census in 2020 (in Japanese). https://www.e-stat.go.jp/en/stat-search/files?page=1&toukei=00200521&tstat=000001136464. Accessed 4 Oct 2023

[15] Economic and social research institute, Cabinet office, Japan (2023) Prefectural accounts [in Japanese]. https://www.esri.cao.go.jp/jp/sna/data/data_list/kenmin/files/contents/main_2019.html. Accessed 4 Oct 2023

[16] Kim HJ, Fay MP, Feuer EJ, Midthune DN (2000) Permutation tests for joinpoint regression with applications to cancer rates. Stat Med 19:335–351 10649300 10.1002/(sici)1097-0258(20000215)19:3<335::aid-sim336>3.0.co;2-z

[17] National cancer institute (2014) Joinpoint help manual 4.9.1.0. https://surveillance.cancer.gov/joinpoint/Joinpoint_Help_4.9.1.0.pdf. Accessed 4 Oct 2023

[18] Hirsch JK, Cukrowicz KC (2014) Suicide in rural areas: an updated review of the literature. J Rural Ment Health 38:65–78

[19] Pearce J, Barnett R, Jones I (2007) Have urban/rural inequalities in suicide in New Zealand grown during the period 1980–2001? Soc Sci Med 65:1807–1819 17618025 10.1016/j.socscimed.2007.05.044

[20] Qin P (2005) Suicide risk in relation to level of urbanicity—a population-based linkage study. Int J Epidemiol 34:846–852 15851392 10.1093/ije/dyi085

[21] Yoshioka E, Hanley SJB, Sato Y, Saijo Y (2021) Geography of suicide in Japan: spatial patterning and rural–urban differences. Soc Psychiatr Psychiatr Epidemiol 56:731–746. 10.1007/s00127-020-01978-7 10.1007/s00127-020-01978-7 PMC8068717 33159535

[22] Ehlman DC, Yard E, Stone DM et al (2022) Changes in Suicide Rates—United States, 2019 and 2020. MMWR Recomm Rep 71:306–312. 10.15585/mmwr.mm7108a5 10.15585/mmwr.mm7108a5 35202357

[23] Borges G, Garcia JA, Pirkis J et al (2022) A state level analyses of suicide and the COVID-19 pandemic in Mexico. BMC Psychiatry 22:460. 10.1186/s12888-022-04095-8 35810285 10.1186/s12888-022-04095-8 PMC9271255

[24] Orellana JDY, de Souza MLP (2022) Excess suicides in Brazil: inequalities according to age groups and regions during the COVID-19 pandemic. Int J Soc Psychiatry 68:997–1009. 10.1177/00207640221097826 35621004 10.1177/00207640221097826

[25] Gerstner RM, Narváez F, Leske S et al (2022) Police-reported suicides during the first 16 months of the COVID-19 pandemic in Ecuador: a time-series analysis of trends and risk factors until June 2021. Lancet Reg Health Am 14:100324. 10.1016/j.lana.2022.100324 35912285 10.1016/j.lana.2022.100324 PMC9310552

[26] Hong J, Knapp M (2013) Geographical inequalities in suicide rates and area deprivation in South Korea. J Ment Health Policy Econ 16:109–119 24327481

[27] Yoshioka E, Hanley S, Sato Y, Saijo Y (2022) Associations between social fragmentation, socioeconomic deprivation and suicide risk across 1887 municipalities in Japan, 2009–2017: a spatial analysis using the Bayesian hierarchical model. BMJ Open 12:e063255. 10.1136/bmjopen-2022-063255 36041759 10.1136/bmjopen-2022-063255 PMC9438050

[28] Arya V, Page A, Spittal MJ et al (2022) Suicide in India during the first year of the COVID-19 pandemic. J Affect Disord 307:215–220. 10.1016/j.jad.2022.03.066 35395323 10.1016/j.jad.2022.03.066 PMC8983610

[29] Gunnell D, Chang S (2016) Economic recession, unemployment, and suicide. In: O’Connor RC, Pirkis J (eds) The international handbook of suicide prevention, 2nd edn. Wiley, Hoboken, New Jersey, pp 284–300

[30] Shand F, Duffy L, Torok M (2022) Can government responses to unemployment reduce the impact of unemployment on suicide? A systematic review. Crisis 43:59–66 33475014 10.1027/0227-5910/a000750

