# Supplementary material for: Temporal evolution of suicide by levels of rurality and deprivation among Japanese adults aged 20 years or over between 2009 and 2022
Source: Soc Psychiatry Psychiatr Epidemiol. 2024 Jul 2;59(11):1909–18. doi: 10.1007/s00127-024-02718-x (PMC11522158; doi:10.1007/s00127-024-02718-x)
Supplement: Supplementary file 1 — Supplementary file1 (PDF 821 KB) [file 127_2024_2718_MOESM1_ESM.pdf]

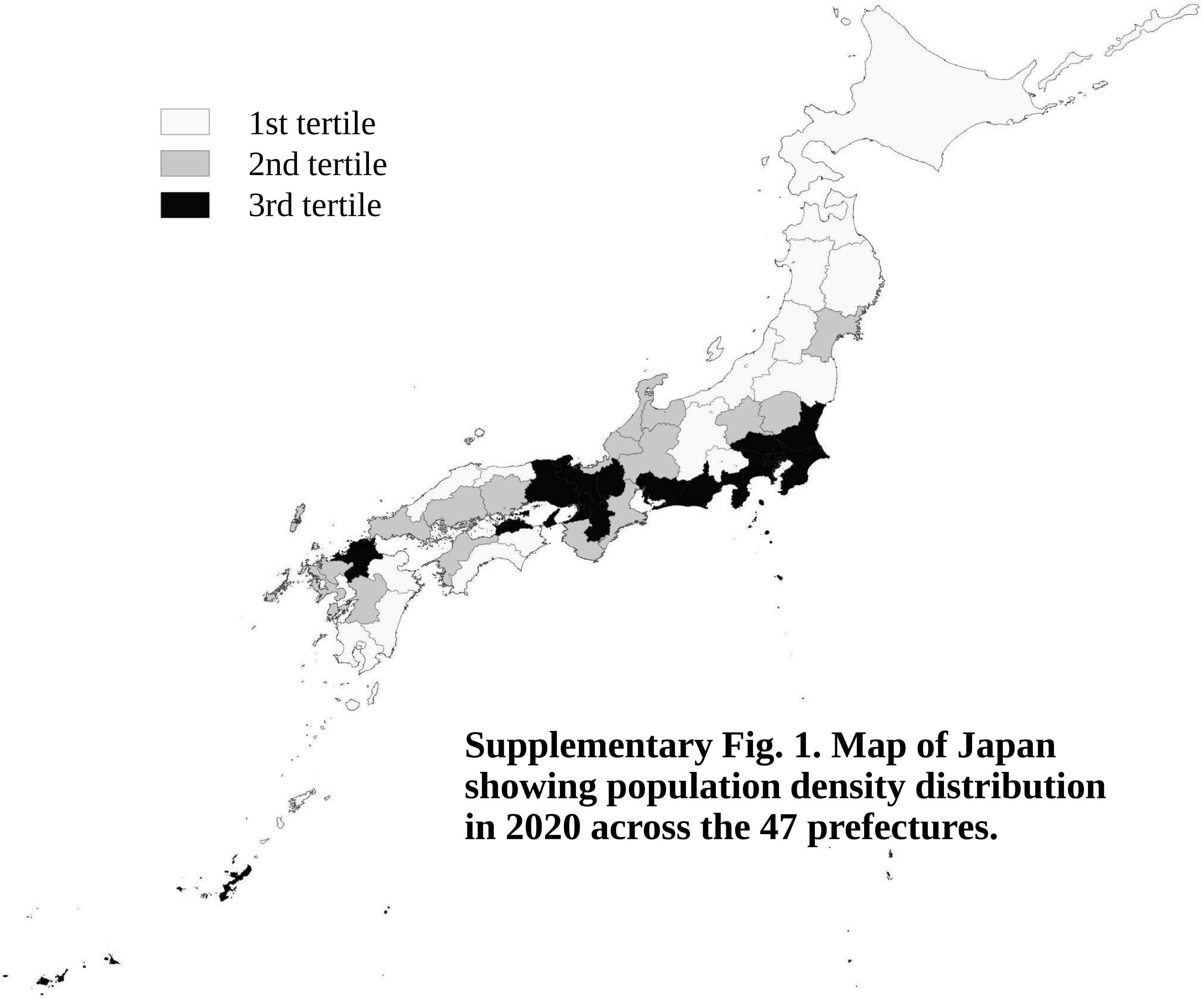

Supplementary Fig. 1. Map of Japan showing population density distribution in 2020 across the 47 prefectures.

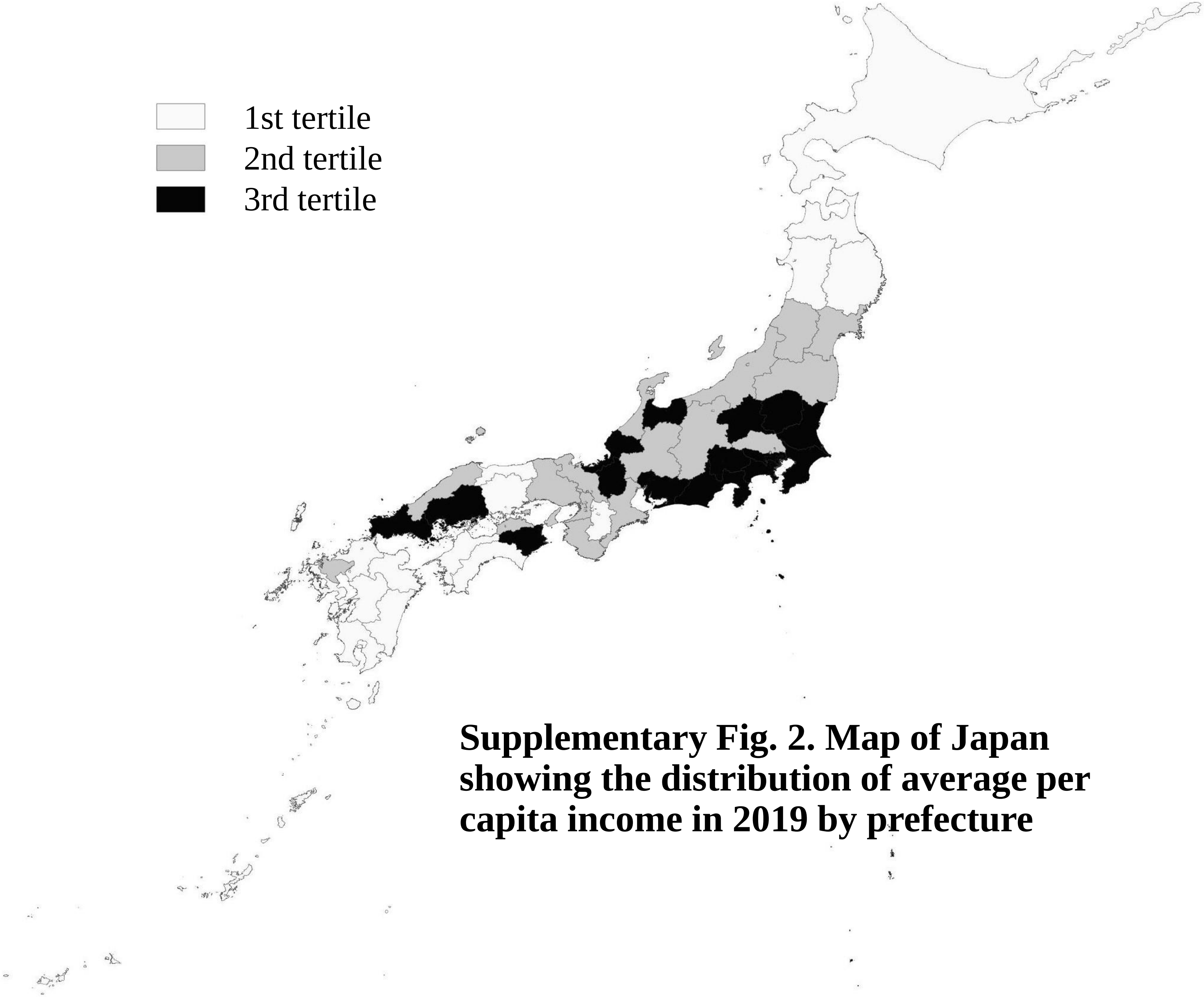

Supplementary Fig. 2. Map of Japan showing the distribution of average per capita income in 2019 by prefecture

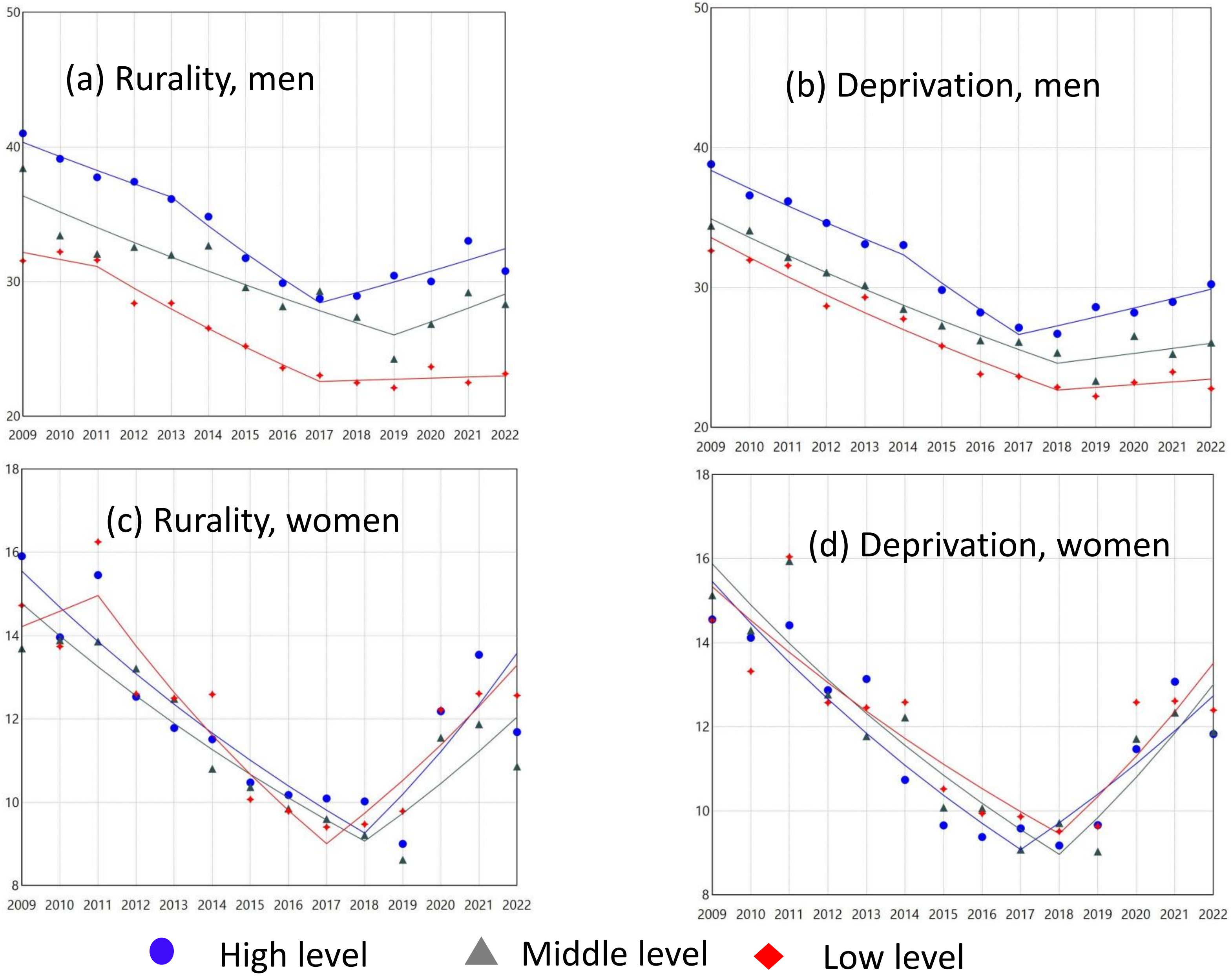

Supplementary Fig. 3. Age-standardized suicide rate per 100,000 population for Japanese men and women aged 20-39 years by levels of rurality and deprivation, 2009–2022, with line segments from joinpoint regression models.

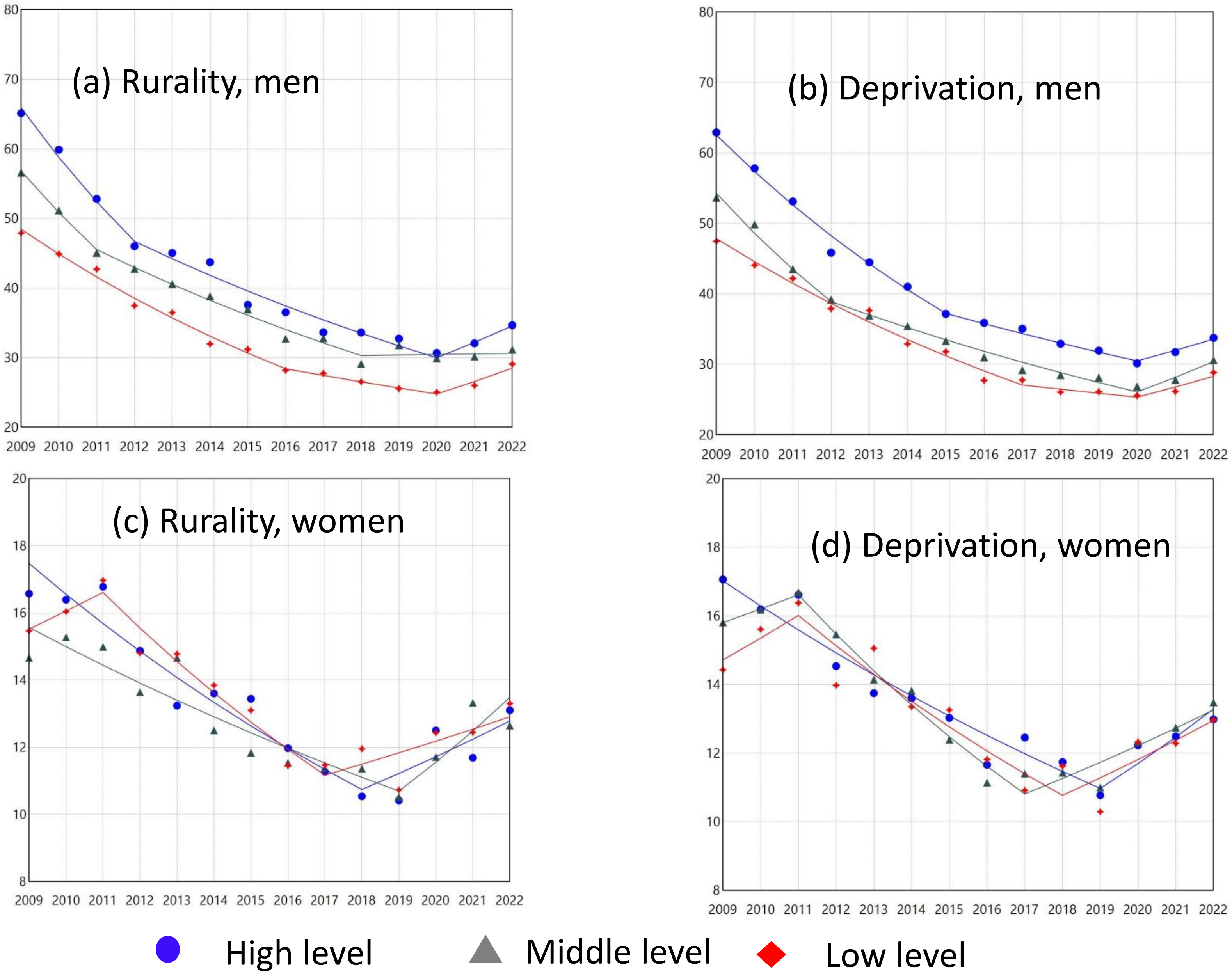

Supplementary Fig. 4. Age-standardized suicide rate per 100,000 population for Japanese men and women aged 40-59years by levels of rurality and deprivation , 2009–2022, with line segments from joinpoint regression models.

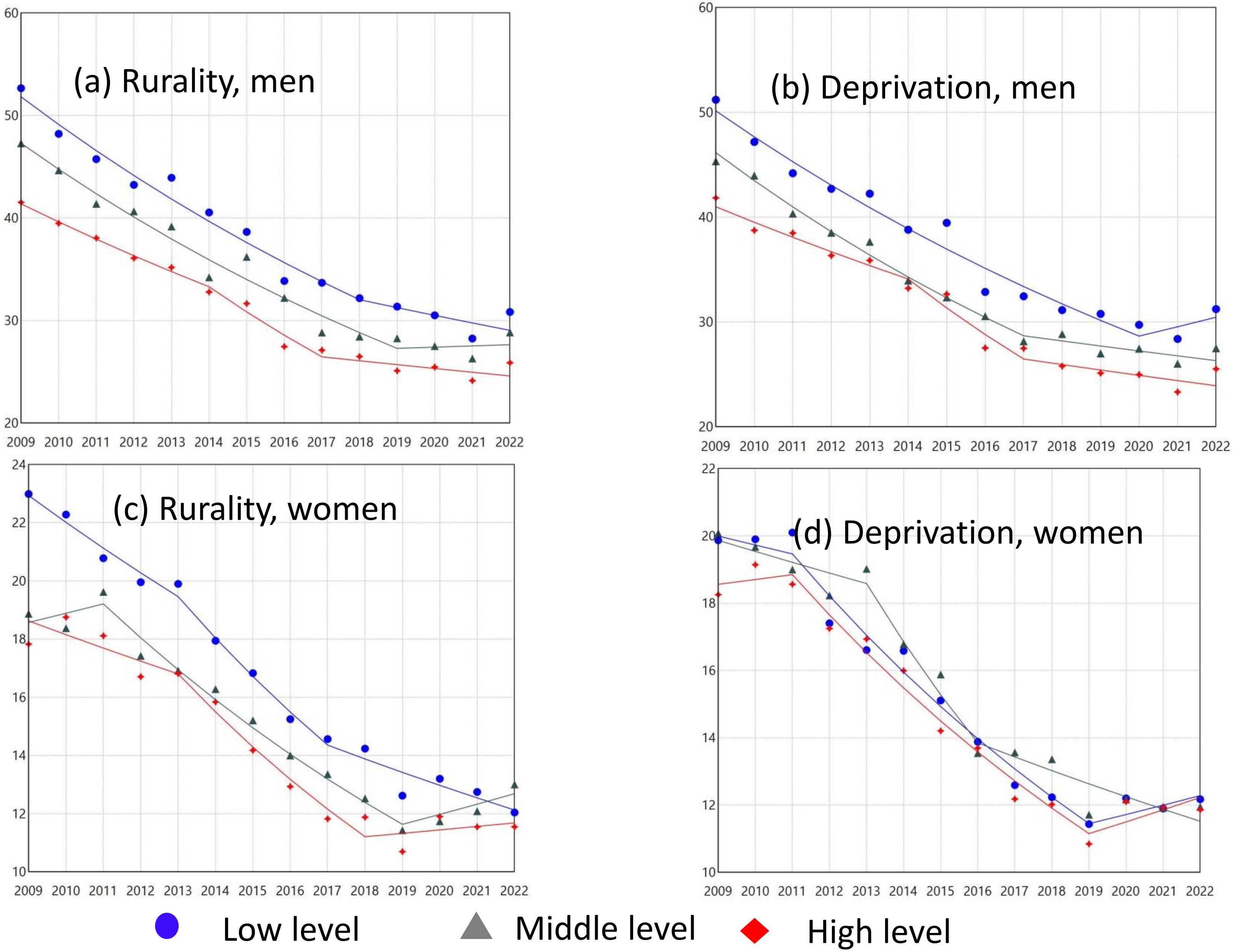

Supplementary Fig. 5. Age-standardized suicide rate per 100,000 population for Japanese men and women aged 60+ years by levels of rurality and deprivation, 2009–2022, with line segments from joinpoint regression models.
