# Supplementary material for: Temporal evolution of suicide by levels of rurality and deprivation among Japanese adults aged 20 years or over between 2009 and 2022
Source: Soc Psychiatry Psychiatr Epidemiol. 2024 Jul 2;59(11):1909–18. doi: 10.1007/s00127-024-02718-x (PMC11522158; doi:10.1007/s00127-024-02718-x)
Supplement: Supplementary file 2 — Supplementary file2 (PDF 261 KB) [file 127_2024_2718_MOESM2_ESM.pdf]

Supplementary Table 1. Number of suicides and suicide rates per 100,000 / population by gender and age in Japan in the years 2009 and 2022.

	2009		2022	
Age	N	Rate	N	Rate
**Men**				
0-19	356	3.0	461	4.5
20-29	2395	32.4	1660	25.5
30-39	3423	36.9	1767	25.4
40-49	3970	48.1	2581	29.3
50-59	5046	60.2	2823	32.1
60-69	4244	49.3	1843	25.1
70-79	2293	40.0	1897	25.1
80+	1297	48.6	1557	34.6
**Women**				
0-19	205	1.8	333	3.4
20-29	1044	14.8	805	13.1
30-39	1326	14.7	760	11.4
40-49	1232	15.1	1051	12.2
50-59	1370	16.1	1236	14.2
60-69	1651	18.0	895	11.7
70-79	1355	19.4	1086	12.3
80+	1100	21.0	929	11.9

Supplementary Table 2. Descriptive statistics of tertiles of prefectural population density in 2020 and per capita prefectural income in 2019, Japan.

	1st tertile		2nd tertile		3rd tertile	
	Median	Range	Median	Range	Median	Range
Population density in 2020 (person per km^2)	135.7	(66.6 to 181.4)	268	(183 to 332.5)	650.5	(351.9 to 6402.6)
Per capita prefectural income in 2019 (Japanese yen)[a]	2704	(2396 to 2838)	2980	(2854 to 3055)	3288	(3058 to 5757)

[a]: One US dollar is worth about JPY 150 in April 2024.

Supplementary Table 3. Summary of Joinpoint regression analysis for trends in suicide rates by rurality and deprivation for Japanese men and women aged 20-39, 2009-2022.

	Segment 1					Segment 2					Segment 3		
	Period	APC	(95%CI)	JP	(95%CI)	Period	APC	(95%CI)	JP	(95%CI)	Period	APC	(95%CI)
**Men**													
Ruraliity [a]													
High	2009-2013	-2.62*	(-3.26 to -1.73)	2013	(2012 to 2014)	2013-2017	-5.92*	(-6.83 to -5.25)	2017	(2017 to 2017)	2017-2022	2.69*	(2.10 to 3.24)
Middle	2009-2019	-3.29*	(-8.34 to -1.42)	2019	(2011 to 2020)	2019-2022	3.75	(-2.64 to 12.64)					
Low	2009-2011	-1.65	(-4.32 to 1.10)	2011	(2011 to 2014)	2011-2017	-5.20*	(-7.24 to -4.13)	2017	(2016 to 2019)	2017-2022	0.37	(-1.05 to 2.43)
Deprivation [b]													
High	2009-2014	-3.36*	(-3.64 to -2.88)	2014	(2013 to 2014)	2014-2017	-6.26*	(-6.79 to -5.46)	2017	(2017 to 2017)	2017-2022	2.32*	(1.85 to 2.75)
Middle	2009-2018	-3.82*	(-4.24 to -3.51)	2018	(2017 to 2019)	2018-2022	1.42	(-0.11 to 3.85)					
Low	2009-2018	-4.27*	(-5.46 to -3.67)	2018	(2015 to 2020)	2018-2022	0.85	(-1.77 to 6.22)					
**Women**													
Rurality [a]													
High	2009-2018	-5,59*	(-7,98 to -4,14)	2018	(2016 to 2020)	2018-2022	10.04*	(3.15 to 26.43)					
Middle	2009-2018	-5,27*	(-8,59 to -3,79)	2018	(2014 to 2020)	2018-2022	7.35*	(0.55 to 22.32)					
Low	2009-2011	2,58	(-4,90 to 11,10)	2011	(2011 to 2014)	2011-2017	-8.11*	(-13.85 to -6.06)	2017	(2016 to 2018)	2017-2022	8.09*	(4.12 to 14.46)
Deprivation [b]													
High	2009-2017	-6.44*	(-13.54 to -4.00)	2017	(2013 to 2020)	2017-2022	7.01*	(0.35 to 26.53)					
Middle	2009-2018	-6.15*	(-8.08 to -4.83)	2018	(2016 to 2020)	2018-2022	9.76*	(3.53 to 23.17)					
Low	2009-2018	-5.23*	(-8.17 to -3.64)	2018	(2015 to 2020)	2018-2022	9.34*	(2.26 to 24.89)					

*: p-value < 0.05. APC: Annual Percentage change. JP: Joinpoint.

[a]: Rurality level was calculated based on the population density in 2020 of the 47 prefectures: the 1st tertile refers to high level, the 2nd refers to middle level, and the 3rd refers to low level.

[b]: Deprivation level was calculated based on the Per capita prefectural income in 2019 of the 47 prefectures: the 1st tertile refers to high level, the 2nd refers to middle level, and the 3rd refers to low level.

Supplementary Table 4. Summary of Joinpoint regression analysis for trends in suicide rates by rurality and deprivation for Japanese men and women aged 40-59, 2009-2022.

	Segment 1					Segment 2					Segment 3		
	Period	APC	(95%CI)	JP	(95%CI)	Period	APC	(95%CI)	JP	(95%CI)	Period	APC	(95%CI)
**Men**													
Ruraliity [a]													
High	2009-2012	-10.82*	(-14.77 to -7.63)	2012	(2011 to 2016)	2012-2020	-5.39*	(-7.23 to -2.91)	2020	(2018 to 2020)	2020-2022	7.35	(-0.86 to 12.06)
Middle	2009-2011	-10.47*	(-11.74 to -8.10)	2011	(2011 to 2012)	2011-2018	-5.65*	(-6.19 to -4.90)	2018	(2017 to 2019)	2018-2022	0.27	(-1.14 to 2.58)
Low	2009-2016	-7.34*	(-7.86 to -7.03)	2016	(2015 to 2017)	2016-2020	-3.35*	(-5.25 to -1.74)	2020	(2019 to 2020)	2020-2022	7.23*	(3.55 to 9.40)
Deprivation [b]													
High	2009-2015	-8.28*	(-10.08 to -7.51)	2015	(2012 to 2017)	2015-2020	-3.92*	(-6.72 to -1.88)	2020	(2018 to 2020)	2020-2022	4.95	(-0.51 to 7.70)
Middle	2009-2012	-10.51*	(-13.86 to -7.72)	2012	(2011 to 2015)	2012-2020	-4.87*	(-6.29 to -2.80)	2020	(2019 to 2020)	2020-2022	7.95*	(2.02 to 11.84)
Low	2009-2017	-6.87*	(-7.89 to -6.38)	2017	(2011 to 2017)	2017-2020	-2.18*	(-7.08 to -1.20)	2020	(2018 to 2020)	2020-2022	5.70*	(1.39 to 8.42)
**Women**													
Ruraliity [a]													
High	2009-2018	-5.26*	(-7.96 to -4.06)	2018	(2014 to 2020)	2018-2022	4.44	(-0.79 to 15.58)					
Middle	2009-2019	-3.68*	(-6.87 to -2.50)	2019	(2014 to 2020)	2019-2022	8.02	(-0.25 to 18.32)					
Low	2009-2011	3.46	(-1.62 to 7.70)	2011	(2011 to 2013)	2011-2017	-6.40*	(-8.85 to -5.47)	2017	(2016 to 2018)	2017-2022	2.93*	(1.12 to 5.53)
Deprivation [b]													
High	2009-2019	-4.29*	(-5.61 to -3.52)	2019	(2017 to 2020)	2019-2022	6.58*	(0.52 to 15.12)					
Middle	2009-2011	2.53	(-1.70 to 5.72)	2011	(2011 to 2012)	2011-2017	-6.91*	(-8.58 to -6.14)	2017	(2016 to 2018)	2017-2022	4.16*	(2.61 to 5.96)
Low	2009-2011	4.30	(-2.43 to 13.01)	2011	(2011 to 2014)	2011-2018	-5.51*	(-10.86 to -4.47)	2018	(2016 to 2020)	2018-2022	4.73*	(0.60 to 14.22)

*: p-value < 0.05. APC: Annual Percentage change. JP: Joinpoint.

[a]: Rurality level was calculated based on the population density in 2020 of the 47 prefectures: the 1st tertile refers to high level, the 2nd refers to middle level, and the 3rd refers to low level.

[b]: Deprivation level was calculated based on the Per capita prefectural income in 2019 of the 47 prefectures: the 1st tertile refers to high level, the 2nd refers to middle level, and the 3rd refers to low level.

Supplementary Table 5. Summary of Joinpoint regression analysis for trends in suicide rates by rurality and deprivation for Japanese men and women aged 60+, 2009-2022.

	Segment 1					Segment 2					Segment 3		
	Period	APC	(95%CI)	JP	(95%CI)	Period	APC	(95%CI)	JP	(95%CI)	Period	APC	(95%CI)
**Men**													
Urbanity [a]													
High	2009-2018	-5.20*	(-8.01 to -4.37)	2018	(2011 to 2020)	2018-2022	-2.42	(-4.69 to 2.10)					
Middle	2009-2019	-5.35*	(-6.11 to -4.89)	2019	(2016 to 2020)	2019-2022	0.44	(-3.03 to 5.97)					
Low	2009-2014	-4.25*	(-4.66 to -3.50)	2014	(2013 to 2014)	2014-2017	-7.37*	(-7.99 to -5.94)	2017	(2017 to 2018)	2017-2022	-1.45*	(-1.98 to -0.58)
SES [b]													
High	2009-2020	-4.96*	(-6.12 to -4.49)	2020	(2014 to 2020)	2020-2022	3.09	(-3.77 to 6.52)					
Middle	2009-2017	-5.77*	(-6.20 to -5.45)	2017	(2016 to 2018)	2017-2022	-1.71*	(-2.63 to -0.40)					
Low	2009-2014	-3.62*	(-4.28 to -2.11)	2014	(2012 to 2014)	2014-2017	-8.09*	(-9.14 to -6.11)	2017	(2017 to 2018)	2017-2022	-2.00*	(-2.99 to -0.03)
**Women**													
Urbanity [a]													
High	2009-2013	-4.03*	(-4.99 to -1.55)	2013	(2011 to 2014)	2013-2017	-7.32*	(-9.10 to -6.08)	2017	(2016 to 2019)	2017-2022	-3.32*	(-4.47 to -0.56)
Middle	2009-2011	1.70	(-2.31 to 5.15)	2011	(2011 to 2013)	2011-2019	-6.08*	(-7.21 to -5.59)	2019	(2018 to 2020)	2019-2022	2.93	(-0.38 to 8.66)
Low	2009-2013	-2.54	(-4.41 to 3.73)	2013	(2011 to 2015)	2013-2018	-7.78*	(-12.07 to -6.25)	2018	(2016 to 2020)	2018-2022	1.05	(-2.18 to 9.01)
SES [b]													
High	2009-2011	-1.33	(-5.09 to 2.71)	2011	(2011 to 2015)	2011-2019	-6.42*	(-9.47 to -5.85)	2019	(2017 to 2020)	2019-2022	2.37	(-1.8 to 8.75)
Middle	2009-2013	-1.66	(-3.07 to 1.55)	2013	(2012 to 2013)	2013-2016	-9.33*	(-10.90 to -6.48)	2016	(2015 to 2019)	2016-2022	-3.02*	(-4.12 to -0.50)
Low	2009-2011	0.77	(-3.71 to 4.70)	2011	(2011 to 2014)	2011-2019	-6.35*	(-8.02 to -5.83)	2019	(2018 to 2020)	2019-2022	3.11	(-0.61 to 9.36)

*: p-value < 0.05. APC: Annual Percentage change. JP: Joinpoint.

[a]: Rurality level was calculated based on the population density in 2020 of the 47 prefectures: the 1st tertile refers to high level, the 2nd refers to middle level, and the 3rd refers to low level.

[b]: Deprivation level was calculated based on the Per capita prefectural income in 2019 of the 47 prefectures: the 1st tertile refers to high level, the 2nd refers to middle level, and the 3rd refers to low level.
